# Genome-Wide Analysis of DNA Methylation and Expression of MicroRNAs in Breast Cancer Cells

**DOI:** 10.3390/ijms13078259

**Published:** 2012-07-03

**Authors:** Sumiyo Morita, Ryou-u Takahashi, Riu Yamashita, Atsushi Toyoda, Takuro Horii, Mika Kimura, Asao Fujiyama, Kenta Nakai, Shoji Tajima, Ryo Matoba, Takahiro Ochiya, Izuho Hatada

**Affiliations:** 1 Laboratory of Genome Science, Biosignal Genome Resource Center, Institute for Molecular and Cellular Regulation, Gunma University, Gunma 371-8512, Japan; E-Mails: msumiyo@gunma-u.ac.jp (S.M.); horii@gunma-u.ac.jp (T.H.); mikimura@showa.gunma-u.ac.jp (M.K.); 2 Division of Molecular and Cellular Medicine, National Cancer Center Research Institute, 5-1-1, Tsukiji, Chuo-ku, Tokyo 104-0045, Japan; E-Mails: rytakaha@ncc.go.jp (R.T.); tochiya@ncc.go.jp (T.O.); 3 Department of Integrative Genomics, Medical Megabank Organization, Tohoku University, 6-3-09, aza Aoba, Aramaki, Aobaku, Sendai 980-8579, Japan; E-Mail: ryamasi@megabank.tohoku.ac.jp; 4 Center for Genetic Resource Information, National Institute of Genetics, Shizuoka 411-8540, Japan; E-Mails: atoyoda@lab.nig.ac.jp (A.T.); afujiyam@lab.nig.ac.jp (A.F.); 5 Human Genome Center, Institute of Medical Science, University of Tokyo, 4-6-1 Shirokanedai, Minato-ku, Tokyo 108-8639, Japan; E-Mail: knakai@ims.u-tokyo.ac.jp; 6 Laboratory of Epigenetics, Institute for Protein Research, Osaka University, 3-2 Yamadaoka, Suita, Osaka 565-0871, Japan; E-Mail: tajima@protein.osaka-u.ac.jp; 7 DNA Chip Research Inc., 1-1-43 Suehirocho, Tsurumi-ku, Yokohama, Kanagawa 230-0045, Japan; E-Mail: matoba@dna-chip.co.jp

**Keywords:** DNA methylation, microRNA, cancer

## Abstract

DNA methylation of promoters is linked to transcriptional silencing of protein-coding genes, and its alteration plays important roles in cancer formation. For example, hypermethylation of tumor suppressor genes has been seen in some cancers. Alteration of methylation in the promoters of microRNAs (miRNAs) has also been linked to transcriptional changes in cancers; however, no systematic studies of methylation and transcription of miRNAs have been reported. In the present study, to clarify the relation between DNA methylation and transcription of miRNAs, next-generation sequencing and microarrays were used to analyze the methylation and expression of miRNAs, protein-coding genes, other non-coding RNAs (ncRNAs), and pseudogenes in the human breast cancer cell lines MCF7 and the adriamycin (ADR) resistant cell line MCF7/ADR. DNA methylation in the proximal promoter of miRNAs is tightly linked to transcriptional silencing, as it is with protein-coding genes. In protein-coding genes, highly expressed genes have CpG-rich proximal promoters whereas weakly expressed genes do not. This is only rarely observed in other gene categories, including miRNAs. The present study highlights the epigenetic similarities and differences between miRNA and protein-coding genes.

## 1. Introduction

DNA methylation plays important roles in development, differentiation, X inactivation, genomic imprinting, and silencing of transposable elements through the regulation of transcription [1–3]. This usually occurs in mammalian cells at cytosine residues in the context of cytosine-phosphate-guanine dinucleotide (CpG), and approximately 60%–90% of cytosines at these sites are methylated [4]. Methyl-CpG serves as the physiological ligand for a family of proteins containing a highly conserved methyl-CpG binding domain (MBD) [5]. These proteins recruit various chromatin-modifying complexes to methyl-CpG sites to cause further chromatin structural changes that result in transcriptional silencing.

Alterations of the patterns of normal DNA methylation result in many human diseases, including cancer [6]. Aberrant patterns of DNA methylation in cancers are associated with tumor type, stage, prognosis, and response to chemotherapy. Human tumors undergo global DNA demethylation, including of Line-1 repetitive elements, and then DNA hypermethylation of certain gene promoters, including those of tumor suppressor genes [7]. Aberrant DNA methylation in cancers is not restricted to protein-coding genes; it is also observed in microRNA (miRNA) genes. MicroRNAs are small noncoding regulatory RNAs of 20–24 nucleotides that reduce the stability and/or translation of fully or partially sequence-complementary target mRNAs. MicroRNAs can act as oncogenes or tumor suppressors, and can contribute to cancer initiation and progression [8].

Genome-wide analysis of DNA methylation is an important issue in epigenetic research. The oldest technology for genome-wide analysis of DNA methylation, Restriction Landmark Genomic Scanning, which utilizes two-dimensional gel electrophoresis of genomic DNA, was developed in 1991 [9,10]. This technology has been used in the cloning of many imprinted genes [10] and tumor suppressors [11,12]. In this century, development of genome-wide analysis technology such as microarrays and next-generation sequencers brought about several new methods for analyzing DNA methylation [13]. Among these methods, capture and next-generation sequencing of methyl-CpG binding domains of MBD1 protein is especially useful for analysis because the binding activity of this protein is stronger than that of antibodies [14].

Although epigenetic silencing of miRNAs has been reported in many cancers, systematic studies of DNA methylation and miRNA transcription have not yet been reported. In addition, differentially methylated regions were found only in the CpG islands just upstream of miRNAs in most reports on the epigenetic silencing of miRNAs because such cases are easily discovered. Therefore, it has not been clarified whether the transcriptional start sites far upstream of miRNAs are influenced by DNA methylation or not. It has also not been clarified whether the CpG-poor promoters of miRNA are influenced by DNA methylation. Furthermore, miRNAs are not only transcribed by RNA polymerase II, which is responsible for most protein-coding genes, but also transcribed by RNA polymerase III. Although the relation between DNA methylation and silencing has been extensively studied in genes transcribed by RNA polymerase II, there are few reports for genes transcribed by RNA polymerase III. Therefore, systematic analyses of the methylation and expression of miRNAs are required. To clarify the relation between DNA methylation and transcription of miRNAs, here we systematically analyzed the methylation and expression of human genes encoding miRNAs, proteins, other non-coding RNAs (ncRNAs), and pseudogenes using next-generation sequencing and microarray analysis.

## 2. Results and Discussion

### 2.1. Genome-Wide Identification of Methylated DNA

To study the genome-wide methylation signature, we performed massive sequencing of methylated DNA enriched by the MBD domain of MBD1, using an Illumina sequencer. We expressed a His-tagged fragment of MBD1 (aa 1–75) in bacteria. This fragment contains the critical MBD domain required for stable and selective binding to methyl-CpG but no structural elements known to contribute to sequence-specific DNA binding [15]. Randomly shared methylated genomic DNA (about 300 bp) was bound to His- and GST-tagged fragments of MBD1 and collected on Dynabeads Talon Magnetic beads, which bind to the His tag. The collected DNA was then purified and sequenced using a next-generation sequencer (Illumina). We performed the genome-wide methylation analysis on a human breast cancer-derived cell line, MCF7, and an adriamycin resistant cancer cell line MCF7/ADR [16]. We obtained 19 million single-end reads for MCF7 and MCF7/ADR with high quality read placement against the human genome.

**Figure 1:**
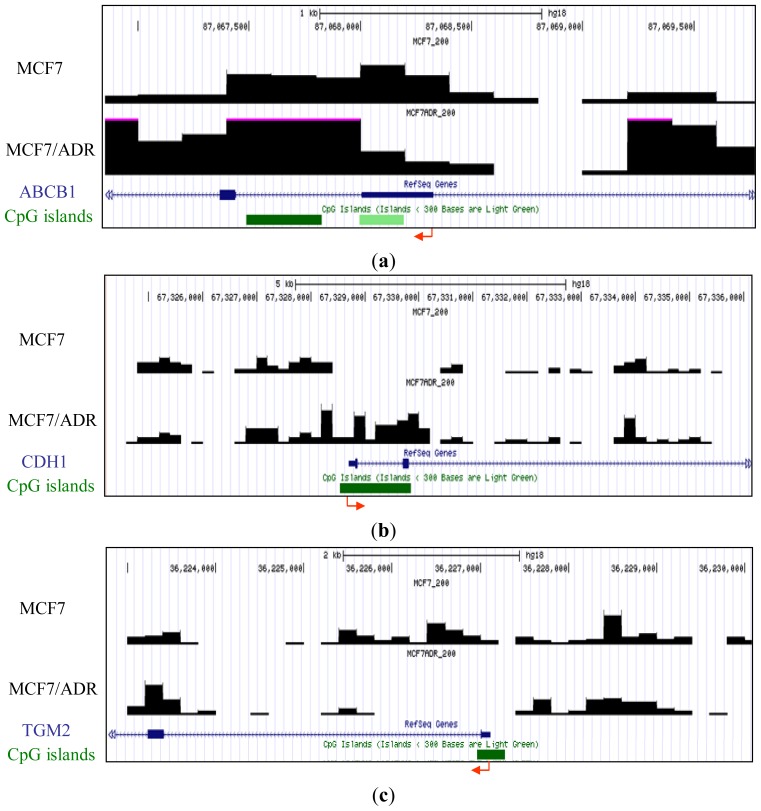
Representative methyl-CpG binding domain (MBD)1DIP-Seq profiles of previously reported differentially methylated promoters between MCF7 and MCF7/adriamycin (ADR). *Y* axis represents the methylation levels of each cell line. Arrows and green bars denote transcription start sites and cytosine-phosphate-guanine dinucleotide (CpG) islands, respectively. (**a**) P-glycoprotein (ABCB1) locus; (**b**) E-cadherin (CDH1) locus; (**c**) transglutaminase 2 gene (TGM2) locus.

To determine the reliability of our genome-wide analysis of DNA methylation, the methylation signature attained in our analysis was confirmed using previous reports. Several genes were reported to be differentially methylated between MCF7 and MCF7/ADR. For example, ABCB1 (MDR1), encoding P-glycoprotein, which is capable of mediating resistance to many antineoplastic drugs commonly used to treat breast cancer by acting as an efflux pump, is hypermethylated in MCF7 but demethylated and overexpressed in drug-resistant MCF7/ADR [17]. We confirmed this differential methylation between MCF7 and MCF7/ADR in our analyzed data (Figure 1a). The silencing of CDH1, which encodes E-cadherin, is important in the epithelial-to-mesenchymal transition. It has been reported by others [18] and confirmed by our results that CDH1 is unmethylated in MCF7 but hypermethylated in MCF7/ADR (Figure 1b). Differential methylation of TGM2 [19], a potential molecular marker for chemotherapeutic drug sensitivity, was also confirmed (Figure 1c).

**Figure 2:**
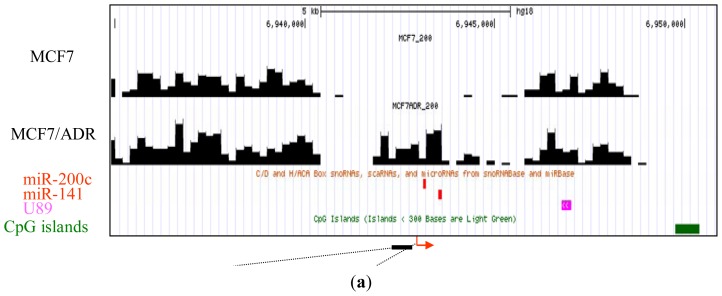

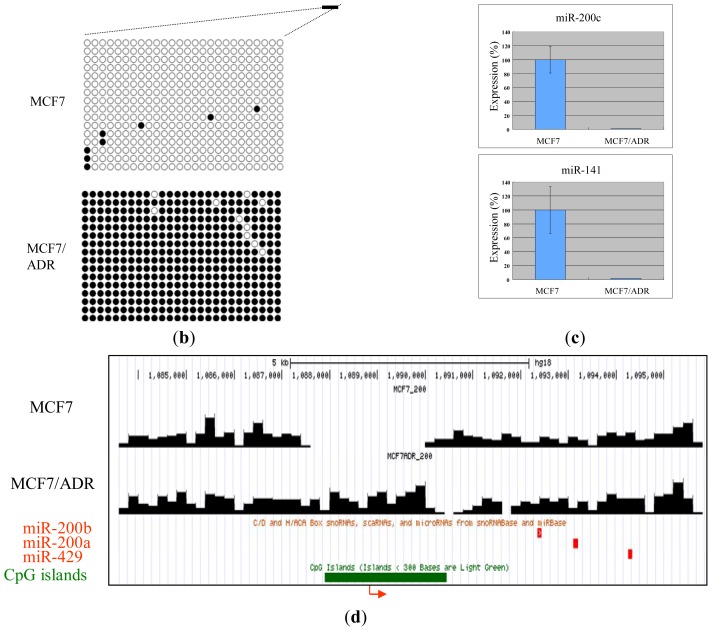
Representative MBD1DIP-Seq profiles of differentially methylated miRNA promoters between MCF7 and MCF7/ADR. Arrows and green bars denote transcription start sites and CpG islands, respectively. Red and magenta blocks indicate pre-miRNAs and scaRNAs, respectively. (**a**) Methylation of the miR-200c/141 locus; (**b**) Bisulfite sequencing results for the differentially methylated promoter of miR-200c/141. The methylation in the region corresponding to the black bar just upstream of the miR-200c/141 transcription start site in (**a**) is presented. Open circles and closed circles denote unmethylated and methylated CpG sites, respectively; (**c**) Real-time-PCR analysis of miR-200c and miR-141 expression. Expression level in MCF7 is normalized as 100%; (**d**) Methylation of the mir-200ab/429 locus.

Next, we compared the methylation around the miRNA promoters between MCF7 and MCF7/ADR and pick up the differentially methylated regions. By confirming these differentially methylated regions using bisulfite sequencing, we tested the reliability of our analysis. Combining nucleosome mapping with chromatin signatures of promoters, 157 proximal promoters of human miRNA [20] were identified and used for the analysis. We found several miRNA clusters, such as miR-200c/141 and miR-200ab/429, which were differentially methylated between these cell lines. For example, the proximal promoter of the miR-200c/141 cluster was hypermethylated in drug-resistant MCF7/ADR (Figure 2a), a finding confirmed by bisulfite sequencing (Figure 2b). In addition, the expression of these miRNAs was downregulated in MCF7/ADR (Figure 2c). Silencing of miR-200 families is important for the maintenance of breast cancer stem cells [21]. This family is also important for the regulation of the epithelial to mesenchymal transition [22] and drug-resistance [23]. The proximal promoter of the miR-200ab/429 cluster was also hypermethylated in MCF7/ADR (Figure 2d), and this was also confirmed to be silenced (data not shown). These results confirmed the reliability of our MBD1-based DNA methylation analysis.

### 2.2. DNA Methylation *versus* Transcription

**Figure 3:**
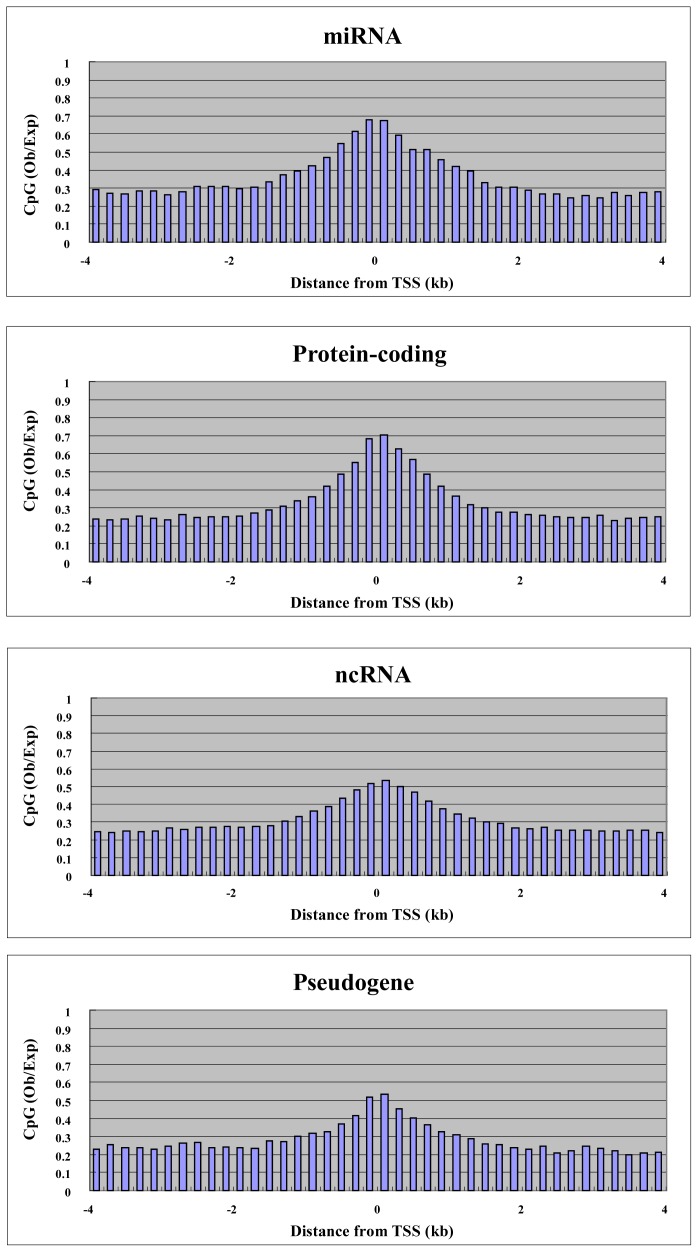
CpG density around the transcription start site for each gene category. The average CpG density is plotted against distance from transcription site.

**Figure 4:**
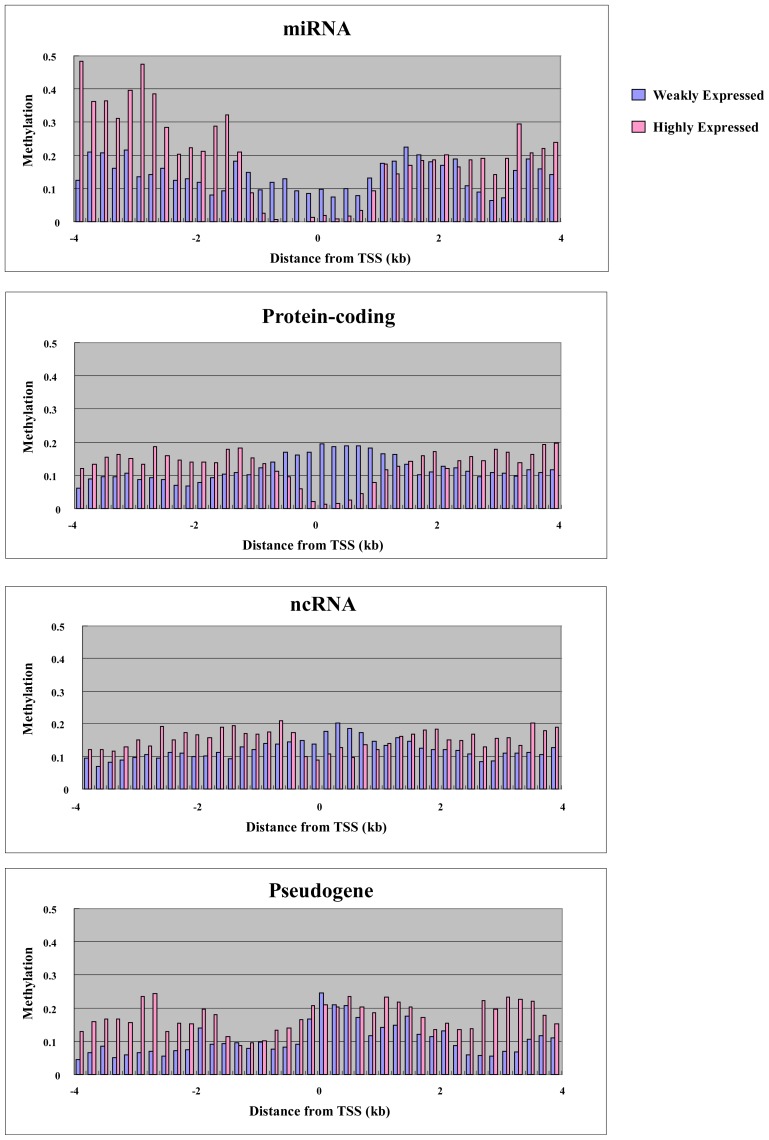
Methylation of weakly expressed and highly expressed genes. The average methylation for each gene category is plotted against distance from transcription start site.

To explore the relationship between gene expression and DNA methylation in the proximal promoter and elsewhere in the gene, we performed microarray analysis using the Agilent platform for miRNA and ordinary genes in MCF7. We were able to use 157 miRNAs, 600 RefSeq non-coding RNAs (ncRNAs) and 235 RefSeq pseudogenes for both expression and methylation analysis. We also used 1000 randomly selected RefSeq protein-coding genes. Before analysis, we plotted the CpG density of each gene category against the position in the genes, since CpG density is an important promoter characteristic. For genes of all categories, the highest CpG density was at the transcription start site, and this is characteristic of promoters (Figure 3). miRNA and protein-coding genes had higher CpG density at the transcription start site compared with other non-coding RNA genes and pseudogenes (Figure 3). We split the genes into two groups: “highly expressed” and “weakly expressed” genes. Highly expressed and weakly expressed genes were defined as those falling within the highest 20% expression quantile and the lowest 20% expression quantile, respectively. For each group, we plotted average cytosine methylation against gene position (Figure 4). This analysis was performed for each category of gene: miRNA, protein-coding, other non-coding RNA, and pseudogene. In the highly expressed genes, we observed low methylation in the proximal promoters for both miRNAs and protein-coding genes (Figure 4). However, low methylation in the highly expressed genes was not observed in the proximal promoters for the other non-coding RNAs and pseudogenes (Figure 4). Thus, DNA methylation in the proximal promoter of miRNAs is tightly linked to transcriptional silencing, as is the case with protein-coding genes.

In addition to proximal promoter methylation, the role of non-promoter methylation, such as in enhancers/far-upstream elements and within the body of the gene, was also examined. Methylation was seen in the gene body and far upstream region, with highly expressed genes showing more extensive methylation compared with weakly expressed genes in all categories, including miRNAs, protein-coding, other non-coding RNAs, and pseudogenes (Figure 4). Thus, non-promoter methylation of miRNAs is linked to transcriptional activation, as with other genes. In mammals, gene-body methylation has been observed in the active human X chromosome when compared with its inactive counterpart [24]. Genome-wide analysis of postnatal neural stem cells indicates that Dnmt3a occupies and methylates non-promoter regions flanking proximal promoters of a large cohort of transcriptionally active genes, many of which encode regulators of neurogenesis [25]. Dnmt3a-dependent nonproximal promoter methylation promotes expression of these neurogenic genes by functionally antagonizing Polycomb repression.

Thus, our genome-wide analysis demonstrated for the first time that the relation between DNA methylation and transcription of miRNAs are similar to that of protein-coding genes although some of miRNAs are transcribed by RNA polymerase III. In agreement with our results, U6 snRNA family genes, which are transcribed by RNA polymerase III, have been reported to be regulated by DNA methylation recently [26]. Increased expression of RNA polymerase III products is often observed in transformed cells [27]. It is also suggested that RNA polymerase III output can substantially affect transformation [27]. Therefore, transcriptional regulation of miRNAs transcribed by RNA polymerase III might play an important role in transformation.

### 2.3. CpG Density *versus* Transcription

**Figure 5:**
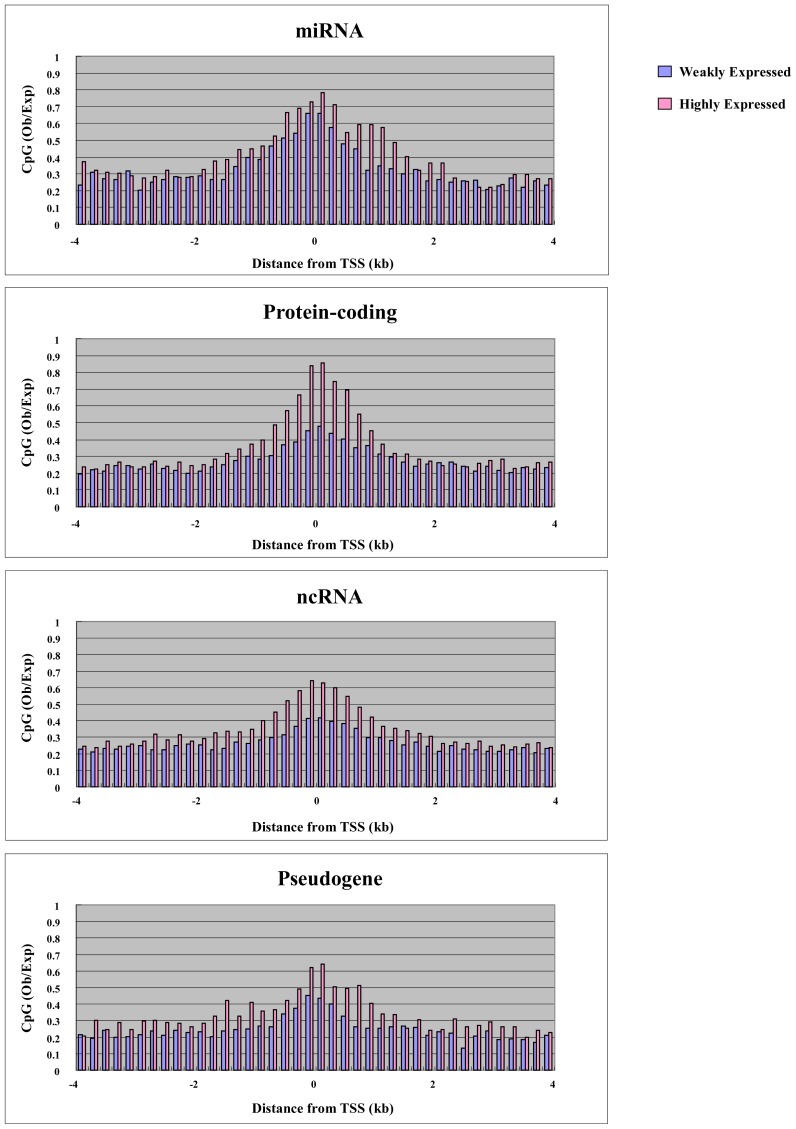
CpG density of weakly expressed and highly expressed genes. The average CpG density of each gene category is plotted against distance from transcription site.

CpG density underlies many characteristics of promoters. Ubiquitously expressed genes have CpG-rich promoters, while tissue-specific genes have CpG-poor promoters [28]. Therefore, it is possible that CpG density can determine the promoter activity of the genes. We plotted the CpG density of highly expressed and weakly expressed genes in each gene category against the position from the transcription start site (Figure 5). In protein-coding genes, highly expressed genes had higher CpG density in the promoter, while weakly expressed genes had lower CpG density (Figure 5). In other words, ubiquitously expressed genes have stronger promoter activity compared with tissue-specific genes. This characteristic is only weakly observed in gene categories other than protein-coding genes (Figure 5). Therefore, this correlation between CpG density and promoter activity could be related to protein-coding ability. Protein-coding genes are transcribed by RNA polymerase II, while genes coding small non-coding RNAs including some miRNAs are transcribed by RNA polymerase III. It is intriguing to think that the transcription by RNA polymerase II is affected by CpG density, while the transcription by RNA polymerase III is not. Non-coding RNAs including small RNA become important in cancer research. Further research is required to explain the relation between CpG density and promoter activity.

## 3. Experimental Section

### 3.1. Cell Lines and Culture Conditions

The human breast cancer-derived cell line, MCF7, and its multi-drug resistant derivative, MCF7/ADR, were cultured in RPMI1640 medium containing 10% FBS.

### 3.2. Isolation of Methyl-DNA by MBD1

Ten micrograms of genomic DNA were sonicated to 100–500 bp using a Bioruptor UCD-250 (Cosmo Bio, Tokyo) sonicator. The capture reaction was performed by adding 10 mg of sonicated DNA to a mixture of 10 micrograms of His-tagged MBD1 fragment (aa 1–75) and 60 μL of Dynabeads TALON in MBD1 buffer (10 mM Tris, pH 7.5, 160 mM NaCl, 0.1% Tween20) on a rotating mixer, overnight, at 4 °C. The beads were washed four times with MBD1 buffer. The bound methylated DNA was then eluted by digestion with Proteinase K in 50 mM Tris (pH 7.5), 10 mM EDTA, 0.5% SDS, at 50 °C for 3 h. Eluted DNA was purified with MinElute spin columns (QIAGEN).

### 3.3. Illumina Genome Analyzer Library Preparation and Sequencing

A single-end library was made following the modified manufacturer’s protocol with reagents supplied in the Illumina DNA sample kit. Briefly, collected methylated DNA was end-repaired using Klenow and T4 DNA polymerases, phosphorylated with T4 polynucleotide kinase, and adenylated using Klenow exo-DNA polymerase, and oligonucleotide adapters were added using DNA ligase. The ligated product was amplified using adapter-specific primers 1.1 and 2.1 with Phusion DNA polymerase using the following protocol:

98 °C (0:30)+[98 °C (0:10)+65 °C (0:30)+72 °C (0:30)]×10 cycles+72 °C (5:00)

Amplified DNA was visualized in a 5% Acrylamide TBE gel, and a 300 to 400 bp size range was excised and purified using SPIN-X cartridges (Costar) and MinElute spin columns (QIAGEN). A single-flow cell lane was sequenced in an Illumina Genome Analyzer. The reds were mapped to the reference human genome sequences (hg18). We normalized the tag counts for each region to tags per million (ppm) in a window size of 200 bases, and this value was represented as methylation level.

### 3.4. Bisulfite Sequencing

One microgram of genomic DNA was bisulfite-treated with the EpiTect Bisulfite kit (QIAGEN) according to the manufacturer’s protocol. PCR amplification was performed on bisulfite-modified DNA using the following primers: TTTATGGTAGGAGGATATATTTGTG and CACCTTAAATCAAACAACTTCAAAC. The resulting PCR product was cloned into a pCR2.1 (Invitrogen) TA-cloning vector and was sequenced using a 3130 DNA sequencer (Applied Biosystems).

### 3.5. Real-Time PCR Analysis of miRNA Expression

Expression of mature miRNAs (miR-200c and miR-141) in human breast cancer cell lines was analyzed by TaqMan miRNA Assay (Applied Biosystems) under conditions defined by the supplier.

### 3.6. Genome-Wide Gene Expression Analysis

Expression analysis of miRNA and the other genes was carried out using Agilent’s microarray-based miRNA and mRNA platforms, respectively. Labeling and hybridization was performed according to the manufacturer’s protocol (Agilent Technologies).

## 4. Conclusions

In this study, we systematically analyzed the methylation and expression of human miRNAs in breast cancer cell lines and compared these levels with those of other gene categories using next-generation sequencing and microarray analysis. We found that DNA methylation in the proximal promoters of miRNAs and protein-coding genes was tightly linked to transcriptional silencing. Expression analysis revealed a correlation between expression level and CpG density of proximal promoters in protein-coding genes; however, this was only weakly observed in miRNA genes. Our observations highlight the epigenetic similarities and differences between miRNA and protein-coding genes.
